# Functional characterization of rare *NRXN1* variants identified in autism spectrum disorders and schizophrenia

**DOI:** 10.1186/s11689-020-09325-2

**Published:** 2020-09-17

**Authors:** Kanako Ishizuka, Tomoyuki Yoshida, Takeshi Kawabata, Ayako Imai, Hisashi Mori, Hiroki Kimura, Toshiya Inada, Yuko Okahisa, Jun Egawa, Masahide Usami, Itaru Kushima, Mako Morikawa, Takashi Okada, Masashi Ikeda, Aleksic Branko, Daisuke Mori, Toshiyuki Someya, Nakao Iwata, Norio Ozaki

**Affiliations:** 1 Department of Psychiatry, Nagoya University Graduate School of Medicine, 65 Tsurumai-cho, Showa-ku, Nagoya, Aichi 4668550 Japan; 2 Department of Molecular Neuroscience, Graduate School of Medicine and Pharmaceutical Sciences, University of Toyama, Toyama, 9300194 Japan; 3 Institute for Protein Research, Osaka University, Osaka, 5650871 Japan; 4 Department of Neuropsychiatry, Okayama University Graduate School of Medicine, Dentistry and Pharmaceutical Sciences, Okayama, 7008558 Japan; 5 Department of Psychiatry, Niigata University Graduate School of Medical and Dental Sciences, Niigata, 9518510 Japan; 6 Department of Child and Adolescent Psychiatry, Kohnodai Hospital, National Center for Global Health and Medicine, Ichikawa, Chiba 2728516 Japan; 7 Department of Psychiatry, Fujita Health University School of Medicine, Toyoake, Aichi 4701192 Japan; 8 Brain and Mind Research Center, Nagoya University, Nagoya, Aichi 4668550 Japan

**Keywords:** *NRXN1*, *N*eurodevelopmental disorder, Autism spectrum disorders, Schizophrenia, Targeted resequencing, Ultra-rare variants, Missense variants, Genotype-phenotype

## Abstract

**Background:**

Rare genetic variants contribute to the etiology of both autism spectrum disorder (ASD) and schizophrenia (SCZ). Most genetic studies limit their focus to likely gene-disrupting mutations because they are relatively easier to interpret their effects on the gene product. Interpretation of missense variants is also informative to some pathophysiological mechanisms of these neurodevelopmental disorders; however, their contribution has not been elucidated because of relatively small effects. Therefore, we characterized missense variants detected in *NRXN1*, a well-known neurodevelopmental disease-causing gene, from individuals with ASD and SCZ.

**Methods:**

To discover rare variants with large effect size and to evaluate their role in the shared etiopathophysiology of ASD and SCZ, we sequenced *NRXN1* coding exons with a sample comprising 562 Japanese ASD and SCZ patients, followed by a genetic association analysis in 4273 unrelated individuals. Impact of each missense variant detected here on cell surface expression, interaction with NLGN1, and synaptogenic activity was analyzed using an in vitro functional assay and in silico three-dimensional (3D) structural modeling.

**Results:**

Through mutation screening, we regarded three ultra-rare missense variants (T737M, D772G, and R856W), all of which affected the LNS4 domain of *NRXN1*α isoform*,* as disease-associated variants. Diagnosis of individuals with T737M, D772G, and R856W was 1ASD and 1SCZ, 1ASD, and 1SCZ, respectively. We observed the following phenotypic and functional burden caused by each variant. (i) D772G and R856W carriers had more serious social disabilities than T737M carriers. (ii) In vitro assay showed reduced cell surface expression of NRXN1α by D772G and R856W mutations. In vitro functional analysis showed decreased NRXN1α-NLGN1 interaction of T737M and D772G mutants. (iii) In silico 3D structural modeling indicated that T737M and D772G mutations could destabilize the rod-shaped structure of LNS2-LNS5 domains, and D772G and R856W could disturb N-glycan conformations for the transport signal.

**Conclusions:**

The combined data suggest that missense variants in *NRXN1* could be associated with phenotypes of neurodevelopmental disorders beyond the diagnosis of ASD and/or SCZ.

## Background

Autism spectrum disorder (ASD) and schizophrenia (SCZ), both of which are highly heritable heterogeneous collections of psychiatric and clinical diagnoses with neurodevelopmental origin [1–3], have been diagnosed based on a codified nosology [4]. It is necessary to clarify the neurobiology underlying these neurodevelopmental disorders, such as central pathophysiology and disease mechanisms. Efforts by the National Institute of Mental Health to resume stalled advancements in the treatment of major psychiatric disorders have led to a reconceptualized research strategy, the Research Domain Criteria initiative, which focuses on constructs of psychology and psychopathology delineated by specific neurocircuitry and molecular entities [5]. Recent advances revealed a complex genetic contribution across neurodevelopmental disorders, as identified in studies of deleterious rare variants such as single-nucleotide variants (SNVs) and intragenic deletions/duplications [6–9]. Whole-exome and whole-genome sequencing have become increasingly feasible as diagnostic testing for patients with nonspecific or unusual disease presentations of possible genetic cause and for patients with clinical diagnoses of heterogeneous genetic conditions [10, 11]. As a result, the enormous number of variants with unknown clinical significance has been detected [12, 13]. Previous studies have largely focused on likely gene-disrupting mutations because it is easy to interpret their contribution. Missense variants, instead, have often been undervalued because of incomplete knowledge.

*NRXN1* (OMIM 600565), located on chromosome 2p16.3, is a well-established risk gene of broad neurodevelopmental disorders [14–16]. Rare exonic deletions overlapping *NRXN1* were first identified in individuals with ASD [17, 18] and intellectual disability (ID) [19]. Subsequently, such deletions have been identified in individuals with various neurodevelopmental disorders. Biallelic variants in *NRXN1* cause Pitt-Hopkins-like syndrome-2 (OMIM #614325), a rare autosomal recessive ID syndrome [20, 21]. Gene-disrupting rare exonic *NRXN1* deletions are estimated to contribute to approximately 0.2% of ASD, ID, and SCZ cases [22, 23]. NRXN1 comprises multiple splice variants of the longer NRXN1α and shorter NRXN1β proteins, both of which function as presynaptic hub adhesion molecules to regulate synapse formation and signaling across the synapse with postsynaptic binding partners including NLGNs, leucine-rich repeat transmembrane neuronal proteins, calsyntenins, and cerebellin precursor protein-glutamate receptor δ complexes [24–29]. Human stem cell models showed *NRXN1* disruption influences synapse function and neuronal connectivity [30, 31]. Such synaptic dysfunction further leads to abnormal behaviors including impaired sensorimotor gating, increased grooming behavior, and impaired nest building and parenting ability in *Nrxn1* knockout mouse models [32, 33]. These models retain construct validity of gene-disrupting variants. Based on human genetic studies, rare missense variants in *NRXN1* have been also linked to broad neurodevelopmental disorders including ASD, SCZ, ID, and seizures [34–36]; however, there are no much studies with functional characterization of SNVs in *NRXN1.* According to the Exome Aggregation Consortium (ExAC) [37], *NRXN1* is defined as a constrained gene with an ExAC missense Z score of 3.02. A positive Z score, particularly a score > 3, indicates that the gene is very intolerant of missense variants.

The purpose of the present study is to characterize rare missense variants in *NRXN1* detected from individuals with ASD and/or SCZ from genes to functional levels to clinical features. Effects of SNVs are considered to be milder than those of intragenic deletions and may be obscured in complex animal models. For example, missense variants in *SHANK3*, the gene mutated in Phelan-McDermid syndrome (OMIM #606232), cause less severe phenotype than exonic *SHANK3* deletion [38–40]. Therefore, we utilized cell-based functional assays and in silico three-dimensional (3D) structural modeling. We combined ASD and SCZ samples in a study cohort for more robust identification of the shared genetic basis of these disorders. To identify putative variants with large effect, we undertook targeted resequencing and a genetic association study of rare coding variants in *NRXN1* in a cohort of 4835 unrelated individuals, followed by phenotypic evaluation of individuals with novel variants. We then performed in vitro functional assay for cell surface expression, NLGN1 binding and/or synaptogenic activity, and in silico three-dimensional (3D) structural modeling of NRXN1 with N-glycan and NLGN1 to determine the impact of the detected variants. Here, we highlight the functional characteristics of missense variants in *NRXN1* on broad neurodevelopmental disorders.

## Methods

### Study samples

Two independent sample sets were used in this study. The targeted-resequencing discovery cohort comprised 192 ASD (mean age ± SD, 16.3 ± 8.4 years; 77.6% male) and 370 SCZ (49.7 ± 4.8 years, 53.0% male). For the genetic association analysis, the case-control sample set comprised 382 ASD (19.6 ± 10.7 years, 77.8% male), 1851 SCZ (46.5 ± 15.1 years, 51.2% male), and 2040 control subjects (44.6 ± 14.7 years, 40.9% male). All participants were unrelated, living on mainland Japan, and self-identified as Japanese. All ASD and SCZ cases fulfilled the criteria listed in the Diagnostic and Statistical Manual of Mental Disorders, Fifth Edition (DSM-5) for ASD or SCZ [4]. Control subjects were healthy volunteers selected from the general population who had no history of mental disorders based on questionnaire responses from the subjects themselves during the sample inclusion step. The study was explained to each participant and/or their parents both verbally and in writing. Written informed consent was obtained from the participants and from the parents for patients younger than 20 years old.

### Screening of variation

Genomic DNA was extracted from peripheral blood or saliva samples using the QIAamp DNA Blood Kit or Tissue Kit (Qiagen, Hilden, Germany) following the manufacturer’s protocol. The next-generation sequencing technology of the Ion Torrent PGM (Thermo Fisher Scientific, Waltham, MA, USA) was used for amplicon resequencing in accordance with the manufacturer’s protocol. We designed custom amplification primers to cover coding exons and flanking intron regions of both *NRXN1α* (Ensembl Transcript ID: ENST00000406316.6, NCBI reference sequences NM_004801 and NP_004792; 1477 amino acids) and *NRXN1β* (Ensembl Transcript ID: ENST00000342183.9, NCBI reference sequences NM_138735 and NP_620072; 442 amino acids) with Ion AmpliSeq Designer (Thermo Fisher Scientific). Sample amplification and equalization were achieved using Ion AmpliSeq Library Kit 2.0 and the Ion Library Equalizer Kit, respectively (Thermo Fisher Scientific). Amplified sequences were ligated with Ion Xpress Barcode Adapters (Thermo Fisher Scientific). Emulsion PCR and subsequent enrichment were performed using the Ion OneTouch Template Kit v2.0 on Ion OneTouch 2 and Ion OneTouch ES, respectively (Thermo Fisher Scientific). Sequence reads were run through a data analysis pipeline of the Ion Torrent platform-specific pipeline software, Torrent Suite version 4.4 (Thermo Fisher Scientific). Read assembly and variant identification were performed by the Ingenuity Variant Analysis software (Qiagen) using FASTQ files containing sequence reads and the Ion AmpliSeq Designer BED file software to map amplicons with default parameters: call quality > 20 and read depth > 10.

### Data analysis

Candidate variants were defined as exonic or splice-site variants with allele frequencies of ≤ 1% in the following two public databases: dbSNP Build 151 [41] and the Genome Aggregation Database (gnomAD) [42]. We then examined two databases as a reference for Japanese controls: Human Genetic Variation Database [43] and integrative Japanese Genome Variation Database [44]. Prediction of significance was performed using PolyPhen-2 [45], MutationTaster [46], Rare Exome Variant Ensemble Learner [47], and Combined Annotation–Dependent Depletion (CADD) v1.5 [48]. Additional clinical variant annotations were obtained from NCBI ClinVar [49] and DECIPHER v9.25 [50]. Localization of a protein domain was based on the Human Protein Reference Database [51]. When available, parents were sequenced to determine inheritance patterns. Evolutionary conservation was assessed using Evola ver. 7.5 [52]. All candidate variants were confirmed by Sanger sequencing with the ABI 3130xl Genetic Analyzer (Thermo Fisher Scientific) using standard methods. Sequence analysis software version 6.0 (Applied Biosystems, Foster City, CA, USA) was used to analyze all sequence data.

### Genetic association analysis

The effective sample size and statistical power were computed using the web browser program, Genetic Power Calculator [53]. An ABI PRISM 7900HT Sequence Detection System (Applied Biosystems) and TaqMan assays with custom probes were used to genotype a putative deleterious variant. Each 384- well plate contained two non-template controls and two samples with the variant. The reactions and data analysis were performed using Genotyping Master Mix and Sequence Detection Systems, respectively, according to standard protocols (Applied Biosystems).

### Phenotypic analysis

We scored the social function of patients with a variant that was possibly associated with ASD and SCZ phenotypes based on variation screening using the Global Assessment of Functioning (GAF). Patients are rated between 0 (most severe) and 90 (least severe) [54]. Clinical features of patients were retrospectively examined from medical records and compared with those of individuals with exonic deletions in *NRXN1* [19, 23, 55, 56]. All comorbidities were diagnosed by experienced psychiatrists according to DSM-5 criteria [4].

### Expression vector construction and recombinant protein expression

The coding sequence of mouse Nrxn1α lacking the signal peptide was cloned into pFLAG-CMV-1 vector (Sigma, St. Louis, MO, USA) to yield pFLAG-NRXN1α. NRXN1α used in this study carried splice segments S1, S2, and S3 but lacked S4 and S5. T737M, D772G, R856W, N790Q, S792A, M735V, M756I, T779M, H845Y, L869M, S743Y, S763C, and R813H mutations were introduced into the pFLAG-NRXN1α vector by PCR-based mutagenesis for the cell surface-expression assay and cell surface-binding assay. Expression vectors for mutated forms of mouse NRXN1α-Fc were generated by PCR-based mutagenesis using pEB6-NRXN1α-ECD-Fc [57] as a template. Fc and NRXN1α-Fc were transiently expressed in Expi293F cells (Thermo Fisher Scientific) using PEI MAX (Polyscience). Culture medium containing 20 μg recombinant proteins was incubated with 200 μg Protein A-conjugated magnetic particles (smooth surface, 4.0–4.5-μm diameter; Spherotech, Libertyville, IL, USA) for the synaptogenic assay.

### Cell surface expression assay

HEK293T cells were maintained in DMEM supplemented with 10% FCS. Expression vectors were transfected into HEK293T cells using PEI MAX (Polyscience, Niles, IL, USA). After 36 h of transfection, cells were incubated with mouse anti-FLAG antibody (1:1000, Sigma) for 1 h followed by fixation with 4% PFA for 20 min and blocking with 10% donkey serum for 1 h. Fixed cells were permeabilized with 0.25% Triton X-100 for 5 min and incubated with rabbit anti-FLAG antibody (1:1000, Sigma) for 1 h. Cell surface and total FLAG-NRXN1α proteins were visualized with Alexa Fluor 488-conjugated donkey anti-mouse IgG (1:500, Thermo Fisher Scientific) and Alexa Fluor 555-conjugated donkey anti-rabbit IgG (1:500, Thermo Fisher Scientific), respectively. Fluorescent images were taken using a confocal microscope (TCS SP5II, Leica, Ernst-Leitz-Strasse, Germany) and fluorescence densities of cells were quantified using the ImageJ 1.37 software (National Institutes of Health, Bethesda, MD, USA). Statistical significance was evaluated by one-way ANOVA followed by post hoc Tukey’s test.

### Synaptogenic assay

Primary cerebral cortical neurons were prepared from mice at postnatal day 0 as described previously [58]. Magnetic beads coupled with Fc or Fc fusion proteins were added to cortical neurons at days in vitro 13 at a density of 5 × 10^4^ beads/cm^2^. After 24 h, cultures were fixed and immunostained with rabbit anti-Shank2 antibody (1:200, Frontier Institute, Ishikari, Japan) followed by Alexa555-conjugated donkey anti-rabbit IgG (1:400, Thermo Fisher Scientific) for confocal microscopy. Quantification of immunostaining signals for Shank2 was performed essentially as previously described [58]. Briefly, Shank2 signal intensities on the beads were measured as the fluorescence mean density within a circle measuring 7 μm in diameter enclosing a coated-bead using the ImageJ 1.37 software. Statistical significance was evaluated by one-way ANOVA followed by post hoc Tukey’s test.

### Cell surface binding assay

Expression vectors for FLAG-tagged wild-type and mutated forms of NRXN1α were transfected into HEK293T cells. Transfected cells were then incubated with Fc and NLGN1-Fc [59] (0.1 μM and 0.03 μM for Fig. 2 and Fig. 4, respectively) in DMEM containing 10% FCS, 2 mM CaCl2, and 1 mM MgCl2 for 30 min at room temperature. NLGN1 used in this study lacked splice segments ssA and ssB. After washing, cells were fixed with 4% PFA, immunostained with mouse anti-FLAG (1:1000, Sigma) and rabbit anti-human IgG (1:2000, Rockland, Gilbertsville, PA, USA) antibodies, and then visualized with Alexa Fluor 555-conjugated donkey anti-mouse IgG and Alexa Fluor 488-conjugated donkey anti-rabbit IgG antibodies (1:400, Thermo Fisher Scientific). HEK293T cells were also transfected with an expression vector for FLAG-tagged NLGN1 and incubated with wild-type or mutated forms of NRXN1α-Fc (0.2 μM) (Fig. S2). After washing and fixing, cells were co-stained with antibodies against FLAG and Fc, followed by incubation with Alexa Fluor dye-conjugated secondary antibodies. HEK293T cell surface FLAG (Alexa Fluor 488) and cell surface-bound Fc (Alexa Fluor 555) signals were imaged using a confocal microscope and fluorescence densities of cells were quantified using the ImageJ 1.37 software. Statistical significance was evaluated by one-way ANOVA followed by post hoc Tukey’s test.

### Western blotting

HEK293T cells were transfected with expression vectors for FLAG-tagged wild-type and mutated forms of NRXN1α using Lipofectamine 2000 transfection reagent (Thermo Fisher Scientific). Two days after transfection, cells were lysed with RIPA buffer. Lysates containing 20 μg protein were separated by sodium dodecyl sulfate-polyacrylamide gel electrophoresis (SDS-PAGE), transferred to polyvinylidene fluoride membranes, and probed with mouse anti-FLAG antibody (1:1000, Sigma) followed by horseradish-peroxidase-conjugated goat anti-mouse IgG antibody (1:2000, Bio-Rad, Hercules, CA, USA). Blots were then developed and imaged using a Luminescent Image Analyzer LAS-4000 mini (Fujifilm, Tokyo, Japan).

### Modeling of the 3D structure

The 3D atomic structure of NRXN1α determined by X-ray crystallography is available as entry 3r05 [60] from the worldwide Protein Data Bank (https://www.wwpdb.org) [61]. Considering protein sorting of NRXN1α, we focused on glycosylation sites. Four putative glycosylation sites (N125, N190, N790, and N1223) are described in the NRXN1α entry (NRX1A_HUMAN) in the Universal Protein Resource (UniProt) [62] by computer predictions. N790 is located in the fourth laminin-neurexin-sex hormone binding globulin (LNS) domain; however, the loop structure 789–792 is missing in PDB entry 3r05; it may be due to high flexibility of the loop with N-glycan. Compensating for the missing region, we built the structure of the four missing residues around N790 (789:CNSS:792) on structure 3r05, using HOMCOS server [63] and Modeller 9.19 [64]. Next, the 3D structure of complex-type N-glycan was built based on the N-glycan structure taken from PDB entry 4fqc [65], as shown in Figure S5. The N-glycan model was attached to N790 and relaxed using the program fkcombu [66]. The details of the procedures are described in Supplementary Methods.

## Results

### Identification of novel variants in *NRXN1*

**Fig. 1:**
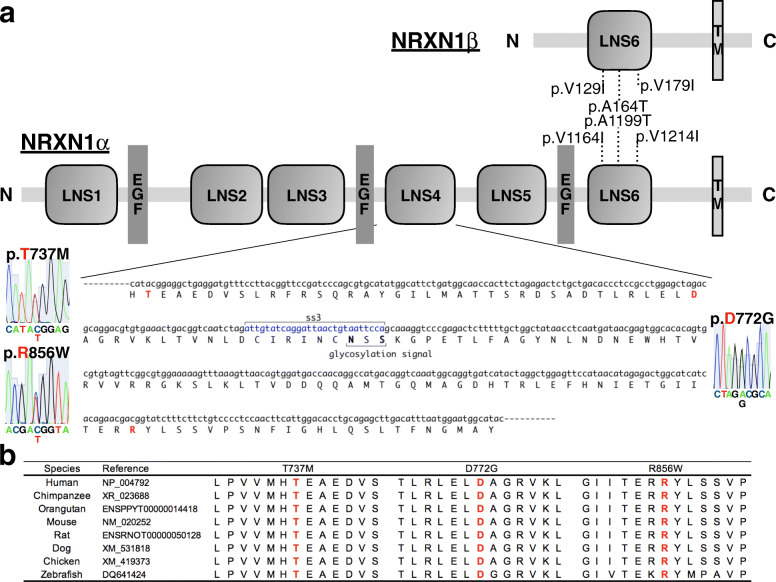
Information about each variant of interest in *NRXN1*. **a** Diagram of NRXN1α and NRXN1β protein (NCBI reference sequences NP_004792 and NP_620072, respectively) with three novel variants detected in this study. NRXN1α contains six LNS domains with three interspersed epidermal growth factor-like (EGF) repeats, followed by an O-linked sugar modification sequence, a short cysteine-loop domain, a transmembrane region, and a cytoplasmic sequence of 55–56 residues. NRXN1β is composed of a unique N-terminal β-neurexin-specific sequence that splices into the NRXN1α sequence N-terminal of its LNS6 domain. Localization of the protein domain is based on the Human Protein Reference Database. LNS, laminin/neurexin/sex hormone binding globulin domain; TM, transmembrane; p, protein. **b** Multiple alignments of amino acid sequences for eight NRXN1α vertebrate homologs

We identified six rare missense SNVs within *NRXN1* coding regions in genomic DNA isolated from Japanese ASD and SCZ subjects (*n* = 562) (Table 1, Fig. 1a). Each variant detected was heterozygous. Nonsense variants, frameshift variants, and splicing-site variants were not found. *NRXN1α* contain six LNS domains with three interspersed epidermal growth factor-like (EGF) repeats, followed by an O-linked sugar modification sequence, a short cysteine-loop domain, a transmembrane region, and a cytoplasmic sequence of 55–56 residues. *NRXN1β* is composed of a unique N-terminal β-neurexin-specific sequence that splices into the *NRXN1α* sequence N-terminal of its LNS6 domain (Fig. 1a) [25, 67]. Of the six missense variants, we regard three SNVs (T737M, D772G, R856W) located within the LNS4 domain of *NRXN1α* as novel because they were classified as damaging in all four in silico prediction tools and because they were present in only two of the public databases. Each of these three SNVs was located in a genomic region that is highly conserved among eight vertebrate species (Fig. 1b). Genomic DNA of the parents was available for three of four subjects carrying these three rare variants. In these three pedigrees, all SNVs were found to be transmitted from a healthy mother (Fig. S1). From the genetic association analysis, all SNVs remained as singleton observations after genotyping for our sample set of cases (*n* = 2233) and controls (*n* = 2040).

**Table 1:** *NRXN1* variants identified in this study

Chr	Position dbSNP ID	Ref	Val	Amino acid variant	Our cohort	iJGVDa	HGVDa	gnomAD^a^	ClinVar	Tools for predicting the deleteriousness of missense variants			
				NP_004792	MAF	MAF	MAF	MAF		PolyPhen-2	MutationTaster	REVEL^b^	CADD^c^
				NP_620072									
2	50091401	C	T	V1214I	1 SCZ	6/7100	4/2420	12/251454	–	0.245	29	0.242	22.1
	rs752722196			V179I	8.9 × 10^− 4^	8.4 × 10 ^− 4^	1.7 × 10 ^− 3^	4.8 × 10 ^− 5^		Benign	Polymorphism		
2	50091446	C	T	A1199T	2 ASD/1 SCZ	17/7086	10/2420	107/282828	Likely benign	0.087	58	0.233	16.98
	rs201336161			A164T	2.7 × 10 ^− 3^	2.4 × 10 ^− 3^	4.1 × 10 ^− 3^	3.8 × 10 ^− 4^		Benign	Polymorphism		
2	50236845	C	T	V1164I	1 ASD	2/7104	1/2054	12/282134	–	0.460	29	0.15	14.39
	rs201881725			V129I	8.9 × 10 ^− 4^	2.8 × 10 ^− 4^	4.9 × 10 ^− 4^	4.3 × 10 ^− 5^		Probably damaging	Polymorphism		
**2**	**50497646**	**G**	**A**	**R856W**	**1 SCZ**	–	–	–	**Uncertain**	**1.0**	**101**	**0.706**	**27.8**
	**rs796052777**			–	**8.9 × 10** ^− **4**^				**Significance**	**Probably damaging**	**Disease causing**		
**2**	**50531259**	**T**	**C**	**D772G**	**1 ASD**	–	–	**1/248460**	–	**1.0**	**94**	**0.761**	**29.6**
	**rs1457374261**			–	**8.9 × 10** ^− **4**^			**4.0 × 10** ^− **6**^		**Probably damaging**	**Disease causing**		
**2**	**50531364**	**G**	**A**	**T737M**	**1 ASD/1 SCZ**	–	–	**2/247276**	**Uncertain**	**1.0**	**81**	**0.686**	**28.4**
	**rs199970666**			–	**1.8 × 10** ^− **3**^			**8.1 × 10** ^− **6**^	**Significance**	**Probably damaging**	**Disease causing**		

Genomic position based on NCBI build GRCh38.p12 (Ensembl Transcript IDs ENST00000406316.6 and ENST00000342183.9). The amino acid position is based on NCBI reference sequences NP_004792 and NP_620072, respectively

*Chr* chromosome, *dbSNP* dbSNP build 151, *Ref* reference, *Val* variant, *MAF* minor allele frequency, *SCZ* schizophrenia, *ASD* autism spectrum disorders, *iJGVD* integrative Japanese Genome Variation Database, *HGVD* Human Genetic Variation Database, *gnomAD* Genome Aggregation Database, *ClinVar* NCBI ClinVar, *PolyPhen-2* polymorphism phenotyping v.2, *REVEL* Rare Exome Variant Ensemble Learner, *CADD* Combined Annotation–Dependent Depletion v1.4

For details of each database, see Supplemental methods. None of the SNVs detected in our study were registered in the DECIPHER database

^a^Minor allele count/total allele count

^b^The REVEL score for an individual missense variant can range from 0 to 1, with higher scores reflecting greater likelihood that the variant is disease-causing

^c^Reference genome SNVs at the 10th-% of CADD scores are assigned to 10, top 1 to 20% and top 0.1 to 30%

### Phenotypic analysis

We examined psychiatric characteristics of individuals with these three *NRXN1* variants. Social impairments were more severe in individuals with D772G and R856W comparing to those with T737M (Table 2).

**Table 2:** Psychiatric characteristics of patients with *NRXN1* SNVs and summary of functional analyses

Variant	T737M	T737M	D772G	R856W
Gender	M	F	M	F
Inheritance	Maternal	Unknown	Maternal	Maternal
Age of evaluation (years)	32	68	9	40
Age at psychosis onset (years)	–	25	–	19
Educational years	16	12	3	12
Marital status	Unmarried	Married with one healthy daughter and two grandchildren	–	Unmarried
Occupation	Desk work with special support	Housewife, part-time worker	Elementary school student (special needs)	–
Hospitalizations	–	–	–	21 years (since her onset)
Neuropsychiatric comorbidity	FIQ 116, depression, ADHD	-	ID, ODD	Treatment-resistant cognitive deficit with continuous delusions
GAF score of evaluation	66	72	33	22
GAF score of lowest ever	35	32	8	1
Cell surface expression	→		**↓**	**↓**
Interaction with NLGN1	↓		**↓**	**→**
Synaptogenic activity	↑		**→**	**→**
Destabilization score of NRXN1 L-shape	1.32		1.93	0.02

*ASD* autism spectrum disorders, *SCZ* schizophrenia, *M* male, *F* female

### Impact of SNVs on membrane localization, synaptogenic activity, and NLGN1 interaction of NRXN1α

**Fig. 2:**
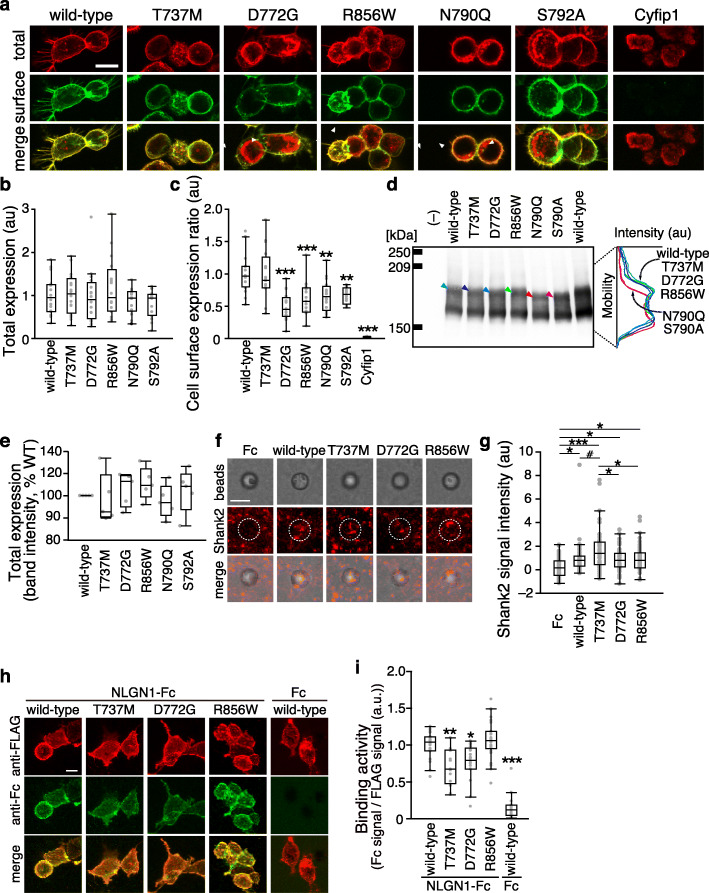
Impact of T737M, D772G, and R856W variants on cell surface expression, synaptogenic activity, and NLGN1 interaction of NRXN1α. **a** Representative images of HEK293T cells expressing wild-type and disease-associated variants of NRXN1α tagged with FLAG epitope. Cell surface and total NRXN1α are shown in green and red, respectively. FLAG-tagged cyfip1, a cytoplasmic protein, serves as a negative control. Arrowheads indicate intracellular accumulation of NRXN1α protein. **b** and **c** Total expression levels (**b**) and ratios of cell surface and total expression levels (**c**) of wild-type and disease-associated variants of NRXN1α in **a** (*n* = 16 HEK293T cells each). **d** Western blot analysis of lysates from HEK293T cells expressing FLAG-tagged NRXN1α variants. Densitographes for each lane are shown on the left. Each densitograph is derived from the lane with an arrowhead of the same color. **e** Total expression levels of FLAG-tagged wild-type and disease-associated variants of NRXN1α measured by band intensity of Western blots in **d** (*n* = 5 independent experiments). Excitatory postsynapse-inducing activities of wild-type and disease-associated (**f**) variants of NRXN1α were monitored by Shank2 immunostaining of co-cultures of cortical neurons and beads conjugated with Fc or NRXN1α variants fused to Fc (middle row, red). Corresponding differential interference contrast images and merged images are shown on the top and bottom rows, respectively. **g** Intensity of staining signals for Shank2 on NRXN1α-Fc beads (*n* = 44–62 beads). **h** Binding of the extracellular domain of NLGN1 fused to Fc to HEK293T cells transfected with FLAG-tagged NRXN1α variants (red). Cell surface-bound Fc fusion proteins were visualized using anti-Fc antibody (green). **i** Ratios of staining signals for NLGN1-Fc and FLAG-tagged NRXN1α variants in **h** (*n* = 13–27 HEK293T cells). Scale bars, 10 μm in **a** and **h**, and 5 μm in **f**. All data are presented as box plots. Horizontal line in each box shows median, box shows the interquartile range (IQR), and the whiskers are 1.5× IQR. #*p* < 0.1, *p < 0.05, **p < 0.01, and ***p < 0.001, Tukey’s test, in comparison with wild-type NRXN1α-expressing cells in **c** and **i**, and in all the comparisons in **g**

We analyzed NRXN1α because each variant detected was located in the LNS4 domain, which only affects the α isoform. Because NRXN1α is a presynaptic membrane protein that regulates synapse organization and specification by interacting with various postsynaptic ligands [29], we investigated the impact of ASD and/or SCZ-associated T737M, D772G, and R856W variants on plasma membrane targeting and synaptogenic activity of NRXN1α. Mouse NRXN1α, which shares more than 99% amino acid sequence identity with human NRXN1α, was used for the functional analyses. Effects of the SNVs on cell surface expression and trafficking were examined in HEK293T cells. N-terminally FLAG-tagged T737M, D772G, and R856W variants of NRXN1α were expressed under the control of the cytomegalovirus promoter. Cell surface and total NRXN1α protein were immunostained with mouse anti-FLAG antibody under non-permeabilized condition and then with rabbit anti-FLAG antibody under cell-permeabilized condition, respectively. Total expression levels of T737M, D772G, and R856W variants of NRXN1α were comparable to that of wild-type NRXN1α (Fig. 2a, b), which was supported by Western blot analysis of whole lysates of the HEK293T cells (Fig. 2d, e). However, relative cell surface expression levels of D772G and R856W variants were significantly lower than that of wild-type NRXN1α (Fig. 2a, c). In fact, intracellular retention of NRXN1α D772G and R856W proteins was detected (arrowheads in Fig. 2a). These results suggest that D772G and R856W substitutions disrupt plasma membrane localization of NRXN1α protein.

We next examined the impact of the SNVs on postsynapse-inducing activity of NRXN1α variants using an artificial synaptogenic assay. In order to evaluate synaptogenic activities of NRXN1α variants, apart from their defects in plasma membrane localization, magnetic beads conjugated with equal amounts of the recombinant extracellular domains of wild-type and variants of NRXN1α were co-cultured with cortical neurons and immunostained for the excitatory postsynaptic scaffold protein Shank2 (Fig. 2f). All disease-associated variants of NRXN1α showed synaptogenic activity as indicated by the accumulation of Shank2 around the beads (Fig. 2f). Excitatory synaptogenic activities were comparable among wild-type NRXN1α, D772G variant, and R856W variant, whereas that of the T737M variant tended to be or was significantly higher than the others (Fig. 2). In contrast, wild-type and variants of NRXN1α used in this study showed no inhibitory postsynapse-inducing activity as monitored by immunostaining for gephyrin (data not shown).

NLGNs are well-known postsynaptic adhesion molecules that interact with NRXN1α [68]. Thus, we examined the effects of T737M, D772G, and R856W variants on binding to NLGN1. HEK293T cells expressing FLAG-tagged NRXN1α variants were incubated with the soluble extracellular domain of NLGN1 fused to Fc and then stained for anti-Fc and anti-FLAG antibodies (Fig. 2h, i). We detected staining signals for NLGN1-Fc on cells expressing wild-type and disease-associated variants of NRXN1α (Fig. 2h). To normalize the differential effects among the variants on the cell surface expression described above, we chose cells with adequate amount of surface expression signals for FLAG-NRXN1α and quantified ratios of cell surface-bound NLGN1-Fc signals and cell surface-expressed FLAG-NRXN1α signals. Fc/FLAG signal ratios were smaller on cells expressing T737M and D772G variants than on those expressing wild-type or R856W variant (Fig. 2i). Consistently, in the cell surface-binding assay of reverse combination in which HEK293T cells expressing FLAG-tagged NLGN1 were incubated with the recombinant extracellular domains of wild-type or mutated forms of NRXN1α fused to Fc, we detected decreased Fc/FLAG signal ratios on cells incubated with recombinant T737M and D772G variants (Fig. S2). These results suggest that T737M and D772G substitutions partly disturb the interaction between NRXN1α and NLGN1.

### Modeling of the 3D structure of SNVs in NRXN1α

**Fig. 3:**
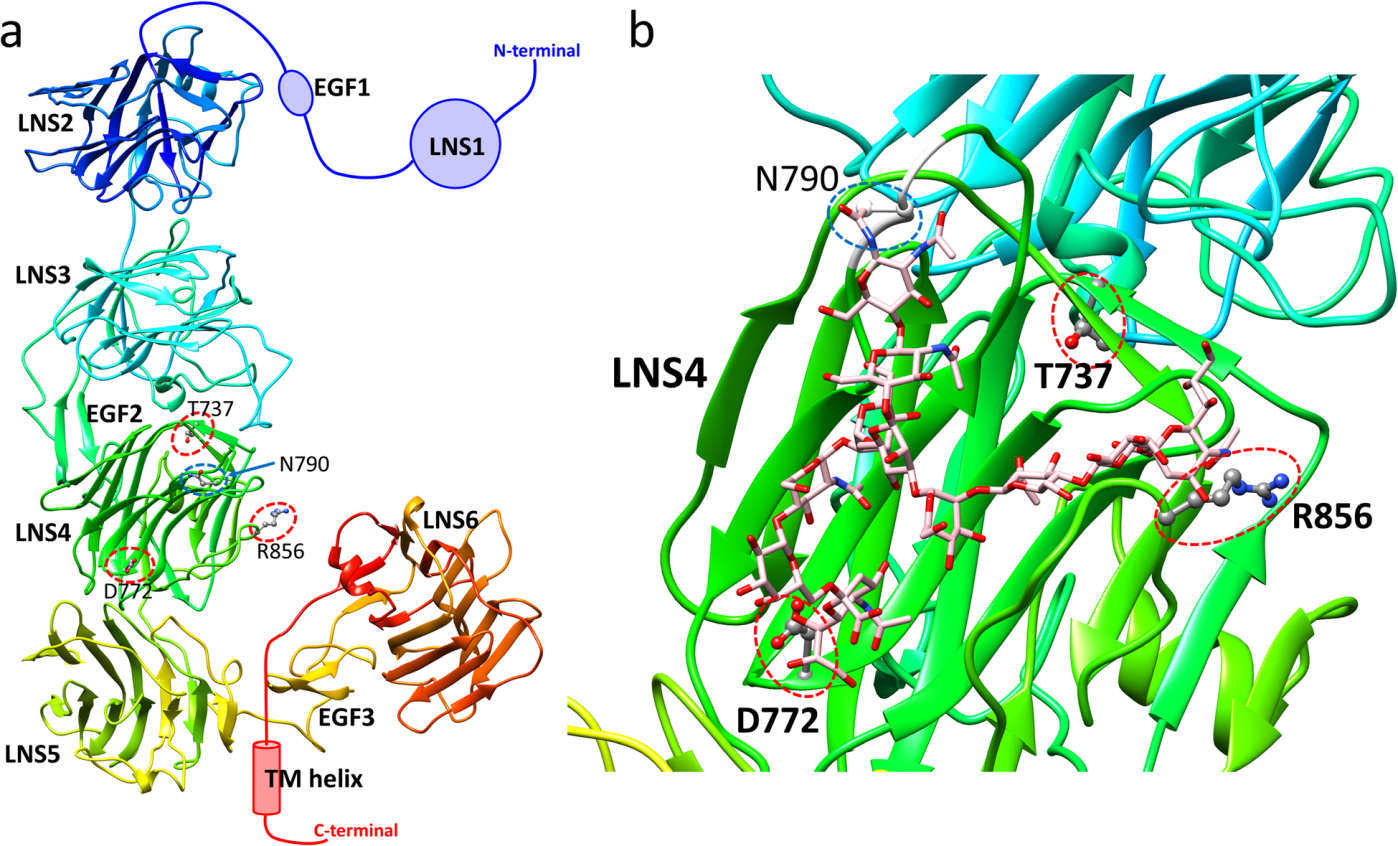
3D structure of NRXN1α (PDB ID: 3r05) with a modeled loop with N-glycan. **a** 3D structure of NRXN1α by ribbon representation. **b** Enlarged view around the LNS4 domain. The three mutated sites (T737, D772, and R856) are indicated by red dotted circles. The potential glycosylation site N790 is enhanced by the blue dotted circle. The model structure for loop 789–792 is indicated by white color. A model structure of complex-type N-glycan is indicated by pink color; this structure is one of the two conformations that contact both D772 and R856 among 300 candidate conformations

The 3D structure of NRXN1α is shown in Fig. 3a. NRXN1α is an L-shaped molecule composed of six LNS domains separated by three interspersed EGF domains. The LNS2-LNS5 domains have a long rod-shaped structure, and EGF3 and LNS6 domains are connected to the rod with a hinge region. Liu et al. [69] also showed the domains LNS2-LNS5 have a rigid linear conformation by electron tomography. All three sites for the novel SNVs (T737M, D772G, R856W) are located in LNS4. An enlarged view around LNS4 is shown in Fig. 3b. The site T737 is buried under the protein surface, whereas sites D772 and R856 are exposed on the surface (Fig. 3b). We also estimated protein stability changes using the program *FoldX* [70] based on the crystal structure (PDB ID: 3r05). The stability changes of T737M, D772G, and R856W are 1.32, 1.93, and 0.02, respectively. Details of the calculation are described in Supplementary information. The calculations suggest that T737M and D772G will destabilize the protein; however, mutation R856W will not largely affect its stability. These results may be because sites T737 and D772 are buried and make hydrogen bonds or salt bridges inside the protein, whereas site R856 is completely exposed to solvents. NRXN1α with mutations T737M or D772G will have the unstable LNS4 domain structure and will not maintain the rigid rod-like shape structure shown in Fig. 3a. If partner molecules, such as NLGN1, interact with the rod shape of NRXN1α, these mutations may disturb the interaction of NRXN1 with its partners.

The complex crystal structure of only the LNS6 domain with NLGN1 is available as PDB entry 3biw [71]. Using the program MATRAS [72], we superimposed LNS6 in 3biw on LNS6 in 3r05 to generate the complex model structure of NRXN1α and NLGN1 (Fig. S3). Interestingly, the superimposed NLGN1 does not significantly clash with NRXN1α and contacts not only with LNS6, but also with LNS4 (Fig. S3a). It also indicates that R856 may interact with NLGN1 (Fig. S3b). This superimposition has been pointed out both by Miller et al. [73] and Chen et al. [60]. This model suggests that LNS4 may interact with NLGN1, although LNS6 provides the primary binding sites for NLGN1. It also implies that mutations in LNS4 may affect the interaction of NRXN1α with NLGN1. The model indicates that R856 may directly interact with NLGN1 (Fig. S3b). The loop corresponding the splice site A of NLGN1 interacts with LNS4 as pointed out by Bourne and Marchot [74]. Because the loop is highly flexible, several 3D models have been built both for Arg and Trp residues at the 856 site of NRXN1α. We found that the interface for the NLGN1 can accept both Arg and Trp by its flexible loop around splice site A (Fig S3b and S3c). These models imply why the mutation R856W did not disturb the interaction between NRXN1α and NLGN1.

Considering the protein sorting of NRXN1α in the membrane transport system, we focused on glycosylation sites. N790 in the LNS4 domain is annotated as a putative glycosylation site in the UniProt database [62]. Because the electron density around the site is missing in the crystal structures, we modeled the structure of the site and other missing three residues with the bound complex-type N-glycan. Among the 300 generated models of N-glycan, 36 models interact with D772 and 13 models interact with R856, but no model was generated where N-glycan interacts with the buried site T737. Six of the 36 models are shown in Figure S4. The model structure shown in Fig. 3b is one of the two models that show N-glycan can interact with both exposed sites D772 and R856. Note that the complex-type N-glycan must have highly flexible conformations; the model shown in Fig. 3b is one of the possible conformations of N-glycan, not a unique stable conformation. However, the models show that N-glycan is long enough to touch the sites D772 and R856 and suggest that their mutations may disturb the conformational ensemble of N-glycan. Based on the 3D models, we generated two hypotheses about how mutations D772G and R856W disturb proper transport to the membrane. First, the mutations may inhibit the glycosylation process of N790. Second, these mutations may disturb the transport signal of N790 glycosylation for packaging the protein into appropriate transport vesicles.

### Impact of NRXN1α SNVs on N790 glycosylation

To address the relationship between D772G and R856W mutations and N790 glycosylation of NRXN1α for proper transport to the plasma membrane, we designed NRXN1α proteins with N790Q and S792A mutations, which should prevent the attachment of N-glycan at N790 and examined cell surface expression levels of these mutants in HEK293T cells (Fig. 2a–c). Relative cell surface expression levels of N790Q and S792A mutant proteins were significantly lower than that of wild-type NRXN1α and were quite similar to those of D772G and R856W variants. In fact, N790Q and S792A mutant proteins expressed in HEK293T cells exhibited slightly faster mobility in SDS-PAGE, indicating that NRXN1α is glycosylated at N790 (Fig. 2d). In contrast, D772G, R856W, and T737M variants showed similar mobility to wild-type NRXN1α in SDS-PAGE (Fig. 2d), and these disease-associated mutations do not seem to affect glycosylation at N790. Therefore, D772G and R856W mutations might disturb the conformation of N-glycan at N790 for packaging into appropriate transport vesicles, although the possibility that these disease-associated mutations and N790 glycosylation mutations independently affect membrane localization of NRXN1α is still not excluded.

Summary of clinical characteristics in individuals with novel variants, in vitro and in silico analyses are shown in Table 2.

### Characterization of other NRXN1α LNS4 variants

**Fig. 4:**
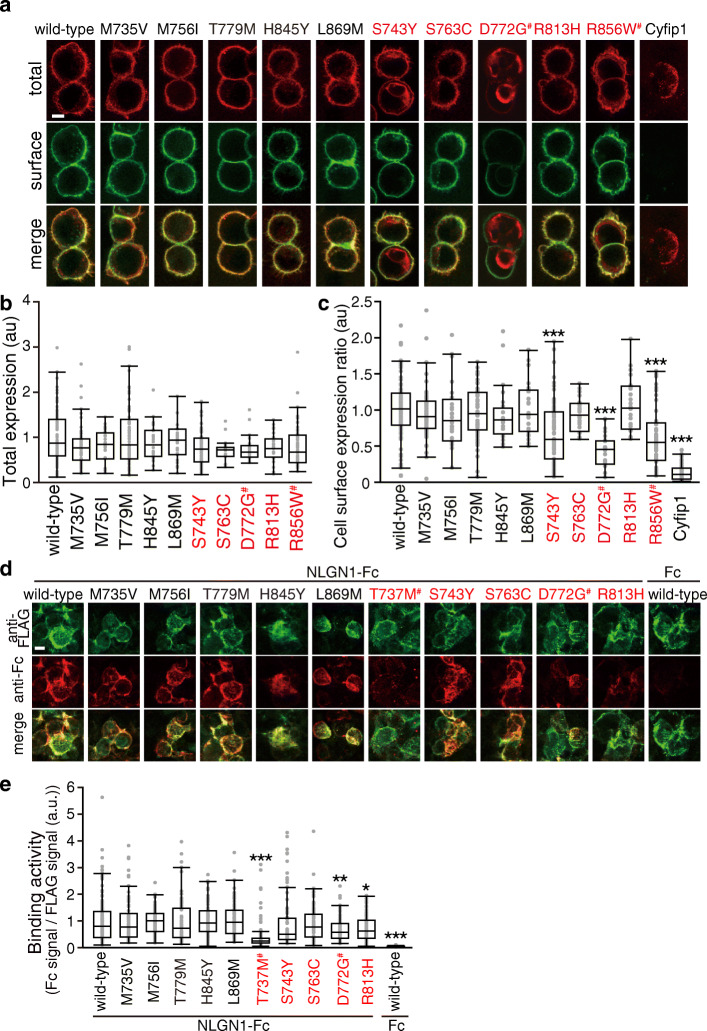
Characterization of NRXN1α variants in LNS4 domain on cell surface expression and NLGN1 interaction. **a** Representative images of HEK293T cells expressing wild-type and disease-associated and non-associated NRXN1α-LNS4 variants tagged with FLAG epitope. Cell surface and total NRXN1α are shown in green and red, respectively. FLAG-tagged cyfip1, a cytoplasmic protein, serves as a negative control. **b** and **c** Total expression levels (**b**) and ratios of cell surface and total expression levels (**c**) of wild-type and LNS4 variants of NRXN1α in **a** (*n* = 24–87 HEK293T cells). **d** Binding of the extracellular domain of NLGN1 fused to Fc to HEK293T cells transfected with FLAG-tagged NRXN1α LNS4 variants (green). Cell surface-bound Fc fusion proteins were visualized using anti-Fc antibody (red). **e** Ratios of staining signals for NLGN1-Fc and FLAG-tagged NRXN1α variants in **d** (*n* = 66–170 HEK293T cells). Scale bars, 10 μm in **a** and **d**. All data are presented as box plots. Horizontal line in each box shows median, box shows the interquartile range (IQR), and the whiskers are 1.5× IQR. *p < 0.05, **p < 0.01, and ***p < 0.001, Tukey’s test compared with wild-type NRXN1α-expressing cells in **c** and compared with wild-type NRXN1α-expressing cells incubated with NLGN1-Fc in **e**. Disease-associated and non-associated variants are colored in red and black, respectively in **b**, **c**, and **e**. #, variants identified in this study

To further evaluate the causal relation between the disrupted membrane localization and NLGN1 binding by these LNS4 missense mutations and etiology of ASD or SCZ, we analyzed eight more LNS4 domain missense variants with equivalent CADD scores. Of the eight SNVs, five were observed with high frequencies in the gnomAD database as control (M735V, M756I, T779M, H845Y, and L869M). Three disease-associated variants were those registered in ClinVar, not in gnomAD (S743Y and S763C), and previously reported as de novo mutation in a case with ASD (R813H) [75] (Table S1). In the cell surface expression assay and NLGN1 binding assay, D772G and R856W variants and T737M and D772G variants were included respectively as positive controls. All the five control variants had no obvious effects on membrane localization and NLGN1 interaction (Fig. 4). In contrast, two out of three disease-associated variants (S743Y and R813H) showed either decreased membrane localization or NLGN1 binding (Fig. 4). We also found both disease-associated variants S743Y and R813H are located on the interface with EGF2 (Figure S6). The interaction between LNS4 and EGF2 may be important both for the cell surface expression and the interaction with NLGN1. These results support the idea that LNS4 domain of NRXN1 is involved in the regulation of membrane localization and NLGN1 binding, the dysregulation of which is associated with the etiology of ASD and/or SCZ.

We summarize in vitro assay and features from 3D models of these variants in Table S2. A correlation between the cell surface expression and the contacts with N-glycan model is observed (Table S3; MCC = 0.386), although three variants (T779M, S763C, and S743Y) are exceptional. The contacts with the 3D bound model of NLGN1 do not correlate with the interaction with NLGN1. It may be due to the flexible loop of NLGN1 accepts both wild type and mutated residues, as shown in Figure S3. Instead of that, the interaction with NLGN1 correlates with the stability change of NRXN1α (Table S4; MCC = 0.463). It implies that the stability of the rod-shape structure of LNS2-LNS5 may be necessary for the interaction with NLGN1.

## Discussion

We performed functional characterization of three ultra-rare missense variants (T737M, D772G, and R856W) within the LNS4 domain of *NRXN1*α isoform, which were regarded as disease-associated variants based on their small fraction registered in public databases (0–2 observations in > 127,000 subjects) and predicted to be protein-damaging by multiple prediction tools mentioned in the “Methods” section (Table 1). Each ultra-rare candidate variant of maternal origin was transmitted to an affected child (Fig. S1), suggesting the variable penetrance. The following phenotypic and functional burden caused by each variant were observed. First, D772G and R856W carriers had more severe functional impairments than T737M carriers. Second, the in vitro assay showed reduced cell surface expression of D772G and R856W mutants, both of which may result from disturbed transport signal associated with N790 glycosylation. Third, in vitro functional analysis showed decreased NRXN1α-NLGN1 interaction with T737M and D772G mutants. Finally, in silico 3D structural modeling indicated that T737M and D772G mutations could destabilize the rod-shaped structure of LNS2-LNS5 domains, and D772G and R856W could disturb N-glycan conformations for the transport signal. The functional significance of the three rare coding variants detected here was supported by additional assays on eight LNS4 variants (five control and three disease-associated variants) with equivalent CADD scores. S843Y, one of the three disease-associated variants, showed a similar decreased membrane localization to D772G, and another variant R813H showed decreased NLGN1 binding like R856W (Fig. 4 and Table 2). The mutated sites of these two variants in LNS4 are on the interface to EGF2 in the in silico model (Fig S6c). Moderate correlations observed between in vitro assays and 3D structure models (Tables S3 and S4) support the validity of the hypothesis proposed in this study.

Reduced cell surface expression of D772G and R856W mutants compared with wild-type and T737M mutant was observed using an in vitro assay. Interestingly, subjects carrying D772G and R856W exhibited severe functional impairments, which are linked to certain rare variants including those in *NRXN1* [3, 76–78]. Because *NRXN1* is one of highly dosage sensitive genes based on NCBI ClinGen Dosage Sensitivity Map [79], our observation of decreased D772G and R856W mutant expression on the plasma membrane might mildly mimic the haploinsufficiency of *NRXN1* deletion. In combination with in silico 3D structural modeling, mutation of D772G and R856W, not T737M, might disturb the transport signal of N790 glycosylation for packaging NRXN1 into appropriate transport vesicles.

Subsequently, we showed increased excitatory synaptogenic activity with T737M mutant only and disturbed NRXN1α-NLGN1 interaction with T737M and D772G mutants. Impairments caused by mutations in the NRXN-NLGN complex have been implicated in the pathomechanisms of not only idiopathic ASD [80] and SCZ [24, 81], but also syndromic ASD, such as Fragile X syndrome [82] and Rett syndrome [83]. Based on the calculation of protein stability changes by mutations, T737M and D772G mutants will not maintain the rod-shape of NRXN1α with destabilization of the LNS4 domain structure; thus, the interaction with NLGN1 may be disturbed. Considering the clinical manifestation of individuals with each SNV, dose-disrupting and destabilized effect on NRXN1 might strongly manifest their phenotype; the treatment resistance of the individual with R856W and early onset disorganized feature of the individual with D772G. Contrary, the discrepancy between increased synaptogenic activity and decreased NLGN1 binding by T737M mutation may be accounted for by a multiple and redundant postsynaptic ligand system for NRXN1 to regulate synaptogenesis [29], which partially explain the milder severity of carriers with T737M.

There are several limitations to this study. First, while there is a rationale for focusing on rare variants within *NRXN1*, the involvement of other genetic factors cannot be ignored. A recent genome-wide study classified ASD and SCZ into different clusters based on over six million common variants [84]. The joint effects of rare variants of large effect and the background of common polygenic variation can be one explanation for the different onset and clinical presentation of two individuals with *NRXN1*-T737M, and the functional similarity of *NRXN1*-D772G with ASD and *NRXN1*-R856W with SCZ, beyond current diagnoses in psychiatry based on subjective reports and clinical observations [85]. Second, regarding genotype-phenotype evaluations, our findings could lead to an additional understanding of the core underlying pathologies and defining subtypes beyond the existing diagnostic classifications; however, we should be careful not to overestimate these results. The contribution of these variants to neurodevelopmental disorders must be quite small because the three variants were not observed in a relatively large sample of individuals with ASD and SCZ. Third, given experience in rare genetic disorders such as Rett Syndrome [83] and Phelan-McDermid syndrome [38–40], it is plausible that both loss of function and missense mutations in *NRXN1* could contribute to risk for neurodevelopmental disorders. More extensive sequencing in the gene would be required rather than targeted sequencing in both cases and controls to determine which variants are relevant. Finally, we only demonstrated the impact of each variant detected on NRXN1 protein function through in vitro functional analysis and in silico 3D structural modeling. In fact, a reduced vesicle release capacity was observed in α-Nrxn 1, 2, and 3 triple knockout mice, whereas a limited reduction in vesicle release capacity was detected in the α-Nrxn2-only knockout mice [27]. Mouse models of Nrxn1 deletion showed abnormalities at the electrophysiological level but did not show major ASD-like behavioral abnormalities such as repetitive behavior or social interaction [32]. NLGN1, 2, and 3 triple knockout mice exhibit little changes in synapse number and expression of postsynaptic scaffold proteins but have severe impairments in synaptic transmission [86]. Together, these data from mouse models suggest that genomic mutations in any of the *NRXN* family genes, as well as the *NLGN* family genes, may be compensatory and suppress the effects of genomic mutations if the remaining genes are normal. With respect to the functional characterization, the spatio-temporal analysis of the effects of molecular network changes caused by *NRXN1* SNVs during development using induced pluripotent stem cell models with knockdown of the variants of interest combined with phenotyping in neuronal cells, or generating conditional mutations in human neurons that are independent of the patients’ genetic background may also be potential avenues to explore.

## Conclusions

Our data from human genetics, in vitro cell biological studies, and in silico informatics characterized *NRXN1* SNVs might link to endophenotypes across neurodevelopmental disorders. As NRXN1 involves an overall transsynaptic signaling network, a more comprehensive approach to address the puzzling diversity of clinical manifestations associated with *NRXN1* SNVs is required. Translation of rare missense variants of disease-causing genes into molecular risk mechanisms to clinical phenotypes is important to advance the clinical utility of human genome sequencing.

## Supplementary information

**Additional file 1 **Supplementary Figures and Tables. **Figure S1.** Family-trio analysis with parental genotype data. **Figure S2.** Decreased NLGN1 binding activities of T737M and D772G variants. **Figure S3.** A modeled 3D complex structure of NLGN1 with NRXN1a. **Figure S4.** Six chosen model structures of LNS4 domain with the complex-type N-glycan among the 300 generated conformations. **Figure S5.** A schematic view of the complex-type N-glycan taken from PDB entry 4fqc. **Figure S6.** Locations of the LNS4 missense mutations in NRXN1a (PDB ID:3r05) with a modeled loop with N-glycan. **Table S1.** Overview of eight control SNVs in NRXN1-LNS4. **Table S2.** Overview of SNVs for in vitro functional assay and 3D models of structures. **Table S3.** Cross tabulation of variants for cell surface expression and N-glycan model. **Table S4.** Cross tabulation of variants for interaction with NLGN1 and stability change.

## Data Availability

Nucleotide sequence data have been submitted to the DNA Data Bank of Japan databases (http://www.ddbj.nig.ac.jp) under the accession number DRA004490. The 3D models have been submitted to the Biological Structure Model Archive (BSM-Arc) under BSM-ID BSM00018 (https://bsma.pdbj.org/entry/18) [87]. The datasets generated and analyzed during the current study are available from the corresponding authors on reasonable request.

## Abbreviations

ASD: Autism spectrum disorder
DSM-5: Diagnostic and Statistical Manual for Mental Disorders, Fifth Edition
EGF: Epidermal growth factor-like
ExAC: Exome Aggregation Consortium
GAF: Global Assessment of Functioning
ID: Intellectual disability
LNS: Laminin-neurexin-sex hormone binding globulin
NLGN1: Neuroligin 1
NRXN1: Neurexin 1
OMIM: Online Mendelian Inheritance in Man
SCZ: Schizophrenia
SNVs: Single-nucleotide variants
UniProt: Universal Protein Resource
3D: Three-dimensional
