# Supplementary material for: Functional characterization of rare NRXN1 variants identified in autism spectrum disorders and schizophrenia
Source: J Neurodev Disord. 2020 Sep 17;12:25. doi: 10.1186/s11689-020-09325-2 (PMC7496212; doi:10.1186/s11689-020-09325-2)
Supplement: Supplementary file 1 — Additional file 1 Supplementary Figures and Tables. Figure S1. Family-trio analysis with parental genotype data. Figure S2. Decreased NLGN1 binding activities of T737M and D772G variants. Figure S3. A modeled 3D complex structure of NLGN1 with NRXN1a. Figure S4. Six chosen model structures of LNS4 domain with the complex-type N-glycan among the 300 generated conformations. Figure S5. A schematic view of the complex-type N-glycan taken from PDB entry 4fqc. Figure S6. Locations of the LNS4 missense mutations in NRXN1a (PDB ID:3r05) with a modeled loop with N-glycan. Table S1. Overview of eight control SNVs in NRXN1-LNS4. Table S2. Overview of SNVs for in vitro functional assay and 3D models of structures. Table S3. Cross tabulation of variants for cell surface expression and N-glycan model. Table S4. Cross tabulation of variants for interaction with NLGN1 and stability change. [file 11689_2020_9325_MOESM1_ESM.pdf]

Additional file 1 for

Functional characterization of rare *NRXN1* variants identified in autism spectrum disorders and schizophrenia

Kanako Ishizuka[1†], Tomoyuki Yoshida[2†], Takeshi Kawabata[3†], Ayako Imai[2], Hisashi Mori[2], Hiroki Kimura[1], Toshiya Inada[1], Yuko Okahisa[4], Jun Egawa[5], Masahide Usami[6], Itaru Kushima[1], Mako Morikawa[1], Takashi Okada[1], Masashi Ikeda[7], Aleksic Branko[1], Daisuke Mori[1,8]*, Toshiyuki Someya[5], Nakao Iwata[7], Norio Ozaki[1]

[†] Contributed equally

*Corresponding author: Daisuke Mori, PhD

Department of Psychiatry, Nagoya University Graduate School of Medicine

65 Tsurumai-cho, Showa-ku, Nagoya, Aichi 466-8550, Japan

Tel: +81 52 7442282; Fax: +81 52 7442293

E-mail: d-mori@med.nagoya-u.ac.jp

Supplementary Methods

Modeling of the loop and N-glycan structure of NRXN1α

Estimation of protein stability changes using the program FoldX

Modeling of the complex structure of NLGN1 with NRXN1α

Supplementary Figures and Table

Figure S1. Family-trio analysis with parental genotype data

Figure S2. Decreased NLGN1 binding activities of T737M and D772G variants

Figure S3. A modeled 3D complex structure of NLGN1 with NRXN1α

Figure S4. Six chosen model structures of LNS4 domain with the complex-type N-glycan among the 300 generated conformations

Figure S5. A schematic view of the complex-type N-glycan taken from PDB entry 4fqc

Figure S6. Locations of the LNS4 missense mutations in NRXN1α (PDB ID:3r05) with a modeled loop with N-glycan

Table S1. Overview of eight control SNVs in NRXN1-LNS4

Table S2. Overview of SNVs for in vitro functional assay and 3D models of structures

Table S3. Cross tabulation of variants for cell surface expression and N-glycan model

Table S4. Cross tabulation of variants for interaction with NLGN1 and stability change

Supplementary Methods

Modeling of the loop and N-glycan structure of NRXN1α

3D atomic structure of bovine NRXN1α determined by X-ray crystallography is available as entry 3r05 [1] from the worldwide Protein Data Bank (wwPDB) (https://www.wwpdb.org) [2]. The structure of human NRXN1α (NRX1A_HUMAN) was modeled using the entry 3r05 as the template. The site N790 is annotated as a potential glycosylation site in the UniProt entry of NRXN1α (NRX1A_HUMAN) [3]. The loop structure 789-792 is missing in PDB entry 3r05; it may be due to high flexibility of the loop with N-glycan. Compensating for the missing region, we built the structure of the four missing residues around N790 (789:CNSS:792) on structure 3r05, using the loop refinement protocol (*loopmodel*) of *Modeller* 9.19 [4] with the help of HOMCOS server [5]. We built 10 different candidate loop conformations with optimizing the conformation only for the loop region 789-792. Next, the 3D structure of the complex-type N-glycan was built based on the N-glycan structure with glycosylated Asn taken from PDB entry 4fqc [6]. One galactose and one sialic acid were manually built, and the fucose was removed as shown in Figure S6. The N-glycan model structure was attached to N790 and relaxed using the program *fkcombu* [7]. The conformation of N-glycan with asparagine was randomly initialized and optimized, and its five atoms (N, CA, C, O and CB) of asparagine were superimposed on the corresponding atoms of N790 of the NRXN1α structure model. And the conformation of the N-glycan was optimized using the steepest descent method using the three energies with equal weights: energy for atom matching, energy for self-clashes and energy for protein-clashes. And the non-specific attraction energy $E_{protatrct}$ between the ligand and the protein also used with the weight = 0.01:

$$E_{protatrct} = -\frac{1}{1+\exp[-\alpha(D_{ar}-R_a-R_r)]} + \frac{1}{1+\exp[-\alpha(D_{ar}-R_a-R_r-\tau\ )]}$$

where D_{ar} is the distance between ligand and protein atoms, R_a and R_p are van der Waals radius of ligand and protein atoms, α is a steepness parameter, and τ is a parameter for tolerance. In this study, we employed $\alpha = 10$ and $\tau = 2$ Å. For every modeled loop conformation among the ten, 30 N-glycan conformations were generated. In total, 300 conformations of N-glycan were generated.

Among the 300 complex-type N-glycan conformations, 47 conformations were close to D772 or R856: 36 were close to D772, 13 were close to R856, and two were close to both D772 and R856. No conformations close to T737 was generated. The criterion of closeness was

that the distance between heavy atoms are less than 6 Å. Figure S4 shows six conformations among the 47 conformations close to D772 or R856. As for the eight LNS4 variants, 95, 36 and 9 conformations were close to T779, S763 and M756, respectively.

Estimation of protein stability changes using the program FoldX

Stability changes by mutations were calculated using the program *FoldX* 4.0 [8]. First, the structure of PDB ID 3r05 was relaxed using *RepairPDB* option of *FoldX*. Next, 20 mutated structures were generated for the three sites T737, D772 and R856 using *BuildModel* option of *FoldX*. The protein stability change was calculated as total energy of mutated residue minus that of wild type residue. A negative value indicates an increased stability, whereas a positive values indicates a decreased stability. The changes of the protein stability for T737M, D772G and R856W were calculated as 1.321, 1.935, and 0.002 kcal/mol, respectively. For the five variants observed in healthy subjects, the changes for M735V, M756I, T779M, H845Y and L869M were 1.100, 1.761, -0.095, -1.592 and -0.606 kcal/mol, respectively. For the three variants detected in cases with neurodevelopmental disorder, the changes for S743Y, S763C and R813H were calculated as 0.744, 1.588 and 1.231 kcal/mol.

Modeling of the complex structure of NLGN1 with NRXN1α

The complex crystal structure of LNS6 domain and rat NLGN1 is available as PDB ID: 3biw. Using the program MATRAS, LNS6 in 3biw was superimposed on LNS6 in 3r05 to generate the complex model structure of NRXN1α and rat NLGN1. Using the complex structure as the template, we modeled human NLGN1 without splice segments ssA and ssB (RefSeq: XP_016861391.1). The loop around ssA was close to R856 of NRXN1α, but a part of the loop (163:EDD:165) was missing in 3biw. The loop conformations of 161:PTEDDIR:167 were built using the loop refinement protocol (*loopmodel*) of *Modeller* 9.19 [4] for both wild type and R856W mutants of NRXN1α. During the loop modeling calculation of NLGN1, the structure of NRXN1α was fixed and regarded as "block" residues. We built 10 different candidate loop conformations, and the two conformations were selected using the DOPE score.

References

1. Chen F, Venugopal V, Murray B, Rudenko G. The Structure of Neurexin 1α Reveals Features Promoting a Role as Synaptic Organizer. Structure. 2011;19:779-89.
2. Berman H, Henrick K, Nakamura H. Announcing the worldwide Protein Data Bank. Nat

Struct Biol. 2003;10:980.

3. The UniProt Consortium. UniProt: the universal protein knowledgebase. Nucleic Acids Res. 2017;45:D158-D69.
4. Sali A, Blundell TL. Comparative protein modelling by satisfaction of spatial restraints. J Mol Biol. 1993;234:779-815.
5. Kawabata T. HOMCOS: an updated server to search and model complex 3D structures. J Struct Funct Genomics. 2016;17:83-99.
6. Mouquet H, Scharf L, Euler Z, Liu Y, Eden C, Scheid JF, Halper-Stromberg A, Gnanapragasam PN, Spencer DI, Seaman MS, Schuitemaker H, Feizi T, Nussenzweig MC, Bjorkman PJ. Complex-type N-glycan recognition by potent broadly neutralizing HIV antibodies. Proc Natl Acad Sci U S A. 2012;109:E3268-77.
7. Kawabata T, Nakamura H. 3D Flexible Alignment Using 2D Maximum Common Substructure: Dependence of Prediction Accuracy on Target-Reference Chemical Similarity. J Chem Inf Model. 2014;54:1850-63.
8. Guerois R, Nielsen JE, Serrano L. Predicting changes in the stability of proteins and protein complexes: a study of more than 1000 mutations. J Mol Biol. 2002;320:369-87.

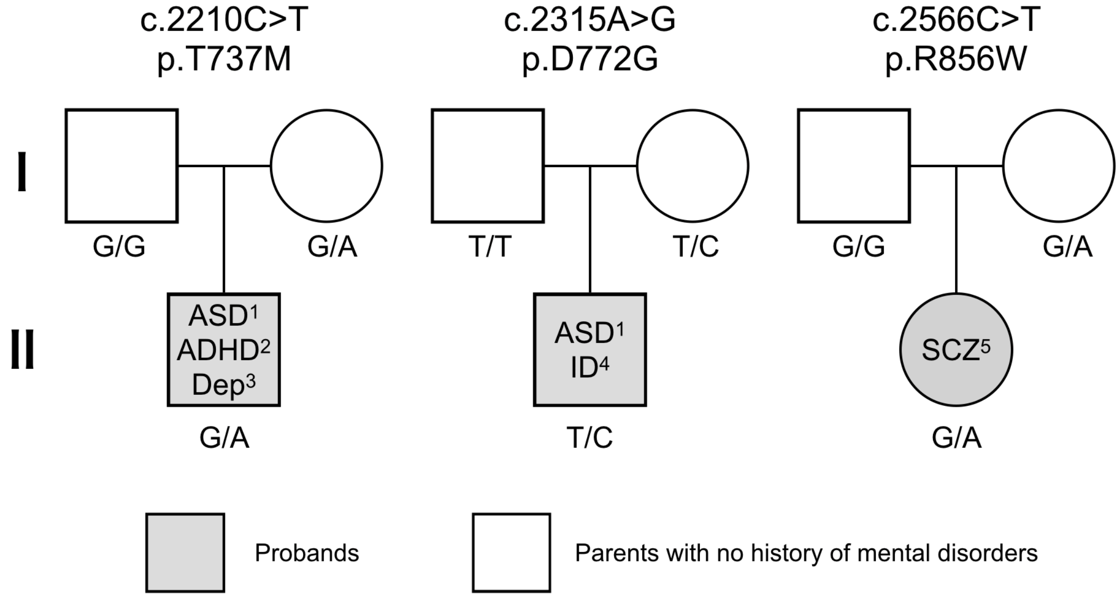

Figure S1. Family-trio analysis with parental genotype data

Note: The genotypes of the tested individuals are indicated on the lower-side.

All comorbidities were diagnosed by experienced psychiatrists according to *Diagnostic and Statistical Manual of Mental Disorders, Fifth Edition* (DSM-5) criteria. c, coding DNA; p, protein [1]Autism Spectrum Disorder; [2]Attention-Deficit/Hyperactivity Disorder; [3]Major Depression; [4]Interectual Disability; [5]Schizophrenia

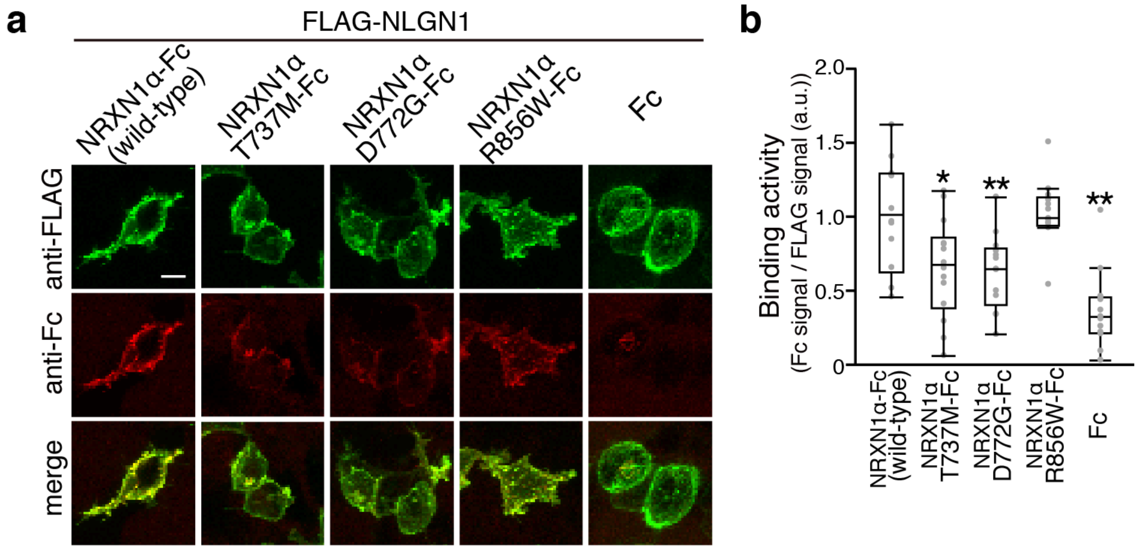

Figure S2. Decreased NLGN1 binding activities of T737M and D772G variants

a. Binding of the extracellular domain of NRXN1α fused to Fc to HEK293T cells transfected with FLAG-tagged NLGN1 (green). Cell surface-bound Fc fusion proteins were visualized using anti-Fc antibody (red). **b.** Ratios of staining signals for NRXN1α-Fc and FLAG-tagged NLGN1 in **a**. (n=13–14 HEK293T cells). Scale bar, 10 μm. All data are presented as box plots. Horizontal line in each box shows median, box shows the interquartile range (IQR) and the whiskers are 1.5× IQR. *$p < 0.05$ and **$p < 0.01$, Tukey's test compared with wild-type NRXN1α-Fc.

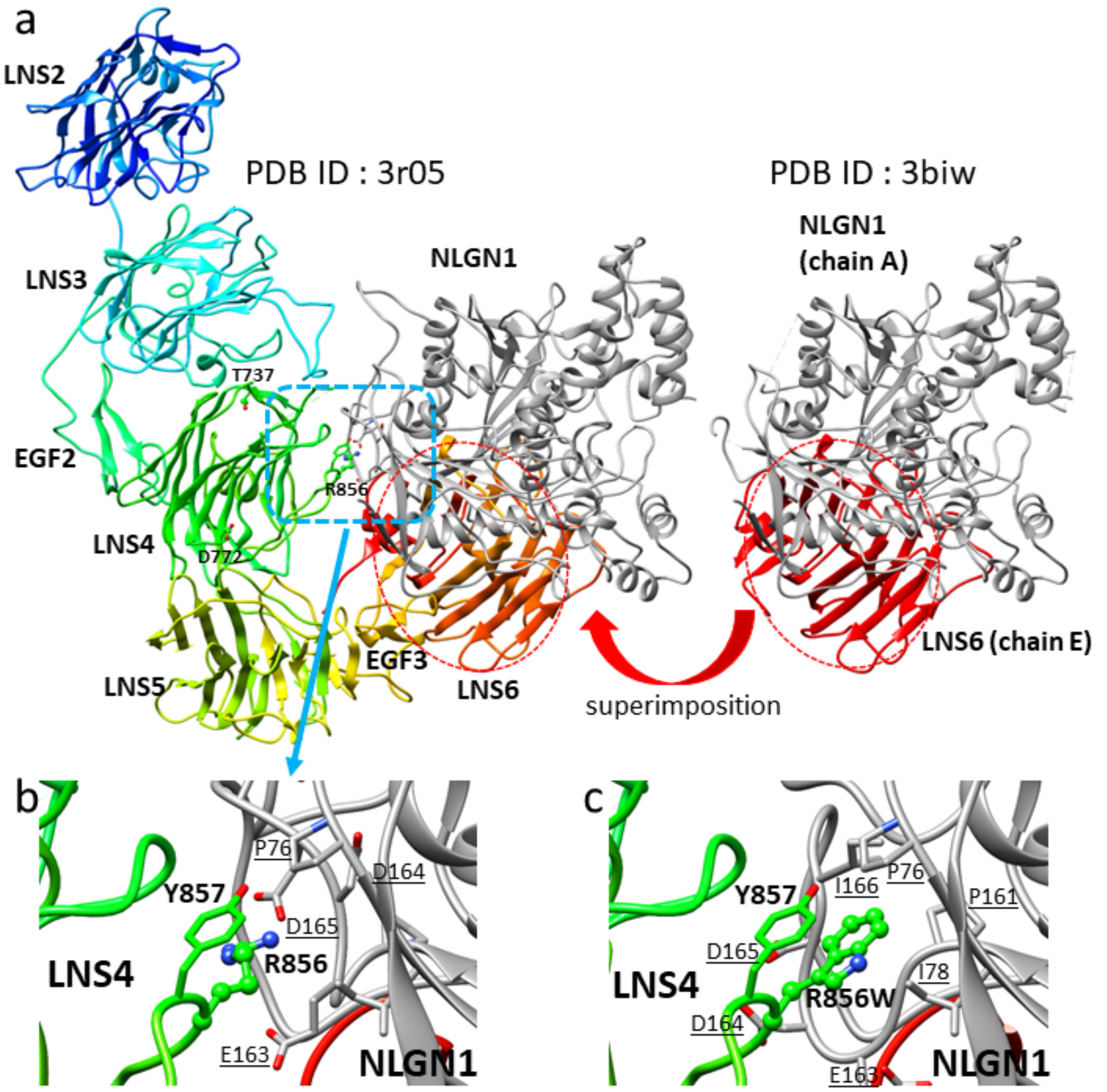

Figure S3. A modeled 3D complex structure of NLGN1 with NRXN1α

a. A complex model structure and the template bound structure. The complex crystal structure of LNS6 domain and rat NLGN1 is available as PDB ID: 3biw. Superimposition of LNS6 in 3biw on LNS6 in 3r05 provides a complex model structure of NRXN1α and NLGN1. The model suggests that NLGN1α interacts not only with LNS6, but also with LNS4. **b**. Enlarged view of the second best loop conformation for the wild type of NRXN1α. R856 forms a salt bridge with D165 of NLGN1. **c**. Enlarged view of the best loop conformation for the R856W mutant of NRXN1α. W856 is in the hydrophobic environment provided by P76, I78, P161, and I166 of NLGN1.

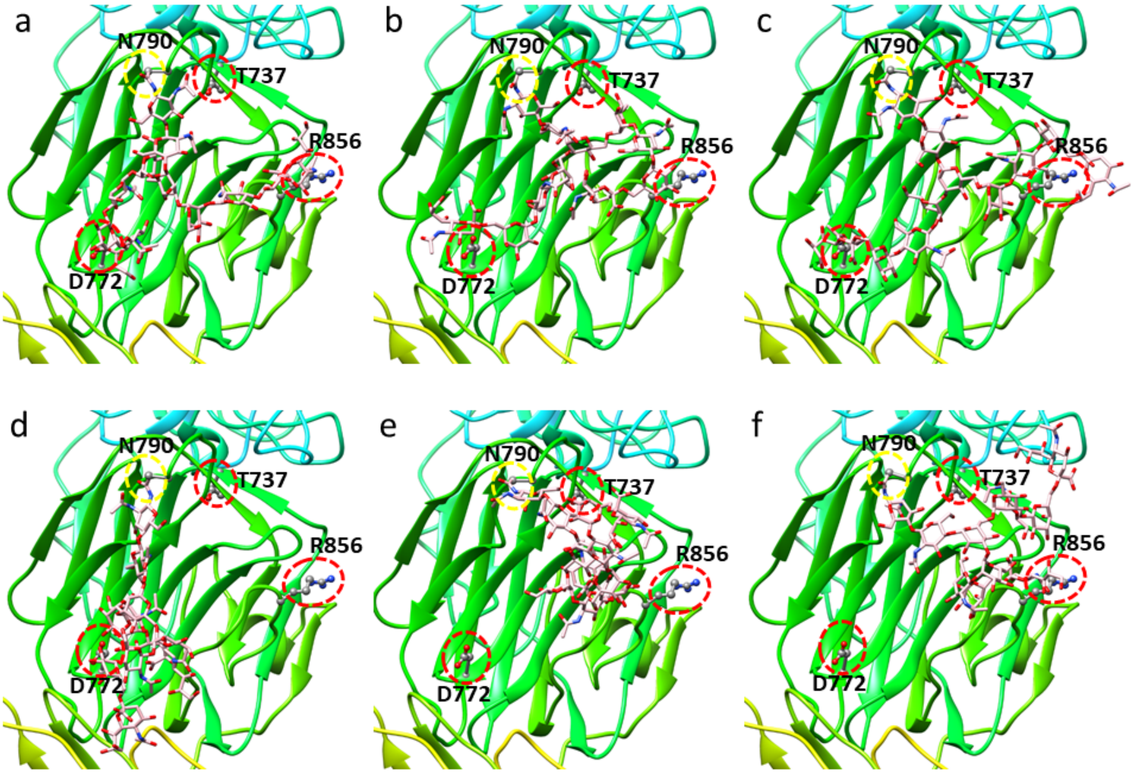

Figure S4. Six chosen model structures of LNS4 domain with the complex-type N-glycan among the 300 generated conformations

The N-glycan conformations a, b, c and d are located within 6 Å from D772. a, b, e and f are located within 6 Å from R856. **a**. 27-th N-glycan for the 2-nd loop conformation. This conformation is also shown in Figure 3b. **b**. 5-th N-glycan for the 2-nd loop conformation. **c**. 26-th N-glycan for the 1-st loop conformation. **d**. 30-th N-glycan for the 1-st loop conformation. **e**. 13-th N-glycan for the 2-nd loop conformation. **f**. 23-th N-glycan for the 7-th loop conformation.

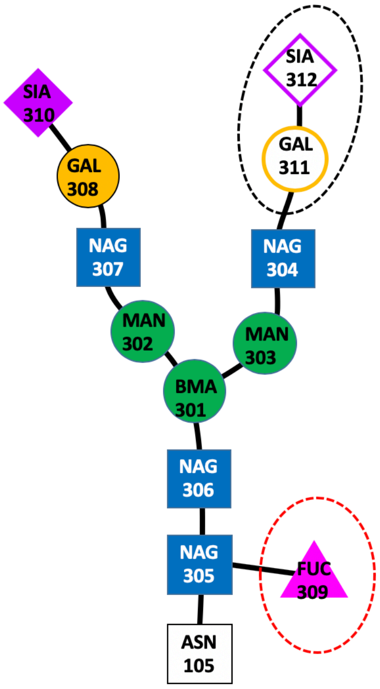

Figure S5. A schematic view of the complex-type N-glycan taken from PDB entry 4fqc

NAG, FUC, BMA, MAN, GAL and SIA stand for N-acetylglucosamine, fucose, b-D-mannose, a-D-mannose, galactose, and sialic acid, respectively. Because GAL311 and SIA312 are missing in the PDB entry, they were modeled manually based on GAL308 and SIA310. The FUC309 was removed.

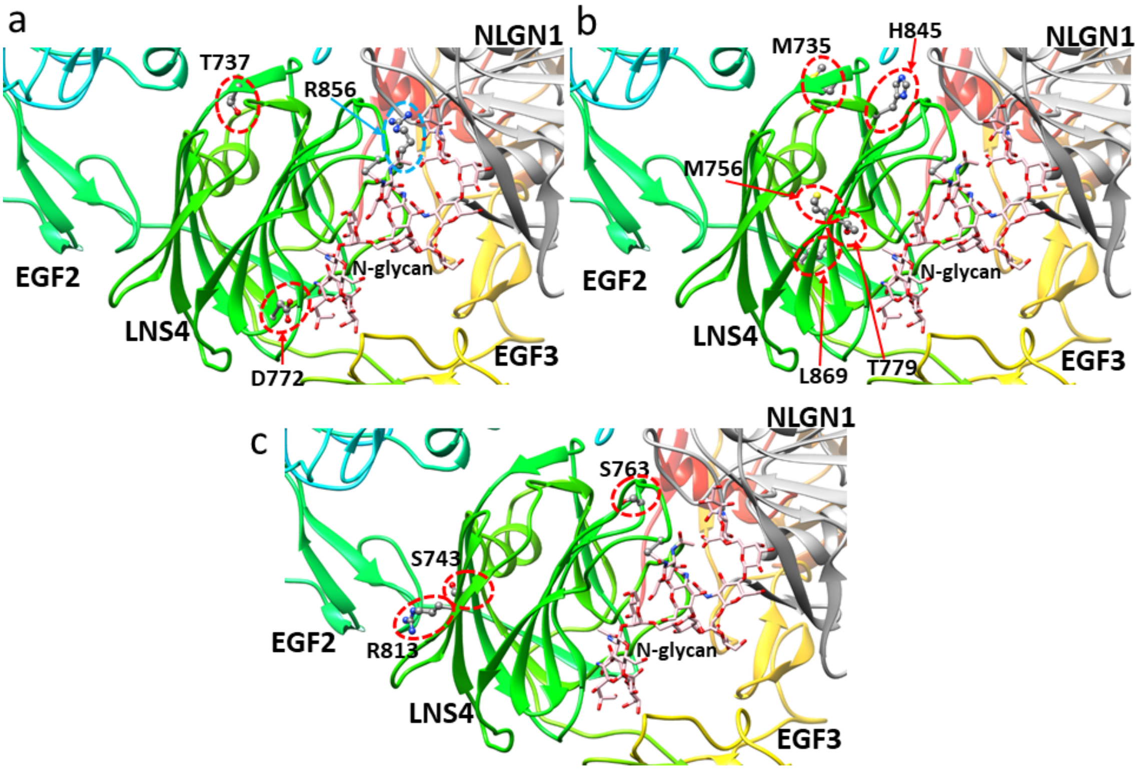

Figure S6. Locations of the LNS4 missense mutations in NRXN1α (PDB ID:3r05) with a modeled loop with N-glycan

a. Three ultra-rare missense sites regarded as disease-related variants (T737M, D772G, and R856W). The variants D772G and R856W showed decreased membrane localization, and T737M and D772G showed decreased NLGN1 binding. The sites D772 and R856 were close to the model of N-glycan. **b**. The sites five missense with equivalent CADD scores registered with high frequencies in gnomAD (M735V, M756I, T779M, H845Y, and L869M). These five variants had no obvious effects on membrane localization and NLGN1 binding (Fig. 4). The site T779 was close to the model of N-glycan. **c**. The sites three disease-associated missense with equivalent CADD scores (S743Y, S763C, and R813H). The variant S743Y showed decreased membrane localization, and R813H showed decreased NLGN1 binding (Fig. 4). The site S763 was close to the model of N-glycan. The sites S743 and R813 interact with the EGF2 domain.

Table S1. Overview of eight control SNVs in *NRXN1*-LNS4

Chr	Position<br>dbSNP ID	Ref	Val	Amino acid variant	gnomAD<br>MAF	ClinVar	CADD[a]
2	50531371<br>rs200622829	T	C	M735V	19/247224<br>7.7×10^{-5}	-	22.4
2	50531306<br>rs201741425	C	T	M756I	20/248466<br>8.0×10^{-5}	-	23.5
2	50531238<br>rs201881725	G	A	T779M	13/247448<br>5.3×10^{-5}	-	26
2	50497679<br>rs200391188	G	A	H845Y	143/279762<br>5.1×10^{-4}	Uncertain significance	25
2	50497607<br>rs201818223	G	T	L869M	212/279966<br>7.6×10^{-4}	Conflicting interpretations of pathogenicity	23.8
2	50531346<br>rs796052774	G	T	S743Y	-	Uncertain significance	28.7
2	50531286<br>rs1558889761	G	C	S763C	-	Uncertain significance	32
2	50506554<br>rs773464948	C	T	R813H<br>*de novo* [1]	9/279772<br>3.2×10^{-5}	-	27.9

Note: Genomic position is based on NCBI build GRCh38.p12. Amino acid position is based on NCBI reference sequence NP_004792.

Ref, reference; Val, variant; gnomAD, Genome Aggregation Database; ClinVar, NCBI ClinVar; MAF, minor allele frequency

[a] Reference genome SNVs at the 10th-% of CADD scores are assigned to 10, top 1% to 20, and top 0.1% to 30.

[1] Reported in the reference 75. O'Roak BJ, Stessman HA, Boyle EA, Witherspoon KT, Martin B, Lee C, et al. Recurrent de novo mutations implicate novel genes underlying simplex autism risk. Nat Commun. 2014;5:5595.

Table S2. Overview of SNVs for *in vitro* functional assay and 3D models of structures

Type of Variant			*In vitro* functional assay		Features from 3D models		
			Cell surface expression	Interaction with NLGN1	Stability change[a]	N-glycan model[b]	NLGN1 model[c]
Reported in this study		T737M		down	1.321		
Reported in this study		D772G	decrease	down	1.935	contact	
Reported in this study		R856W	decrease		0.022	contact	contact
Registered with high frequencies in gnomAD		M735V			1.100		
Registered with high frequencies in gnomAD		M756I			1.761		
Registered with high frequencies in gnomAD		T779M			-0.095	contact	
Registered with high frequencies in gnomAD	Registered in ClinVar	H845Y			-1.592		contact
Registered with high frequencies in gnomAD	Registered in ClinVar	L869M			-0.606		
Not listed in gnomAD	Registered in ClinVar	S743Y	decrease		0.7435		
Not listed in gnomAD	Registered in ClinVar	S763C			1.588	contact	
De novo finding		R813H		down	1.231		

Note: gnomAD, Genome Aggregation Database; ClinVar, NCBI ClinVar

[a] stability changes of NRX1α by mutations were calculated by *FoldX*. A negative value indicates an increased stability, whereas a positive value indicates a decreased stability.

[b] 3D structure models of N-glycan are shown in Figures 3, S4 and S6.

[c] 3D structure models of NRXN1α and NLGN1 are shown in Figures S3 and S6.

Table S3. Cross tabulation of variants for cell surface expression and N-glycan model

MCC=0.386[a]		Cell surface expression	
		Decrease	No change
Contact with N-glycan model	Contact	**D772G**, **R856W**	T779M, S763C
	No contact	S743Y	**T737M,** H845Y, M735V, L869M, M756I, R813H

Note: [a] *MCC i*s Matthews correlation coefficient, as a measure of two-class classifications. It is defined as follows:

$$MCC = \frac{N_{11}N_{00} - N_{10}N_{01}}{\sqrt{(N_{11} + N_{10})(N_{11} + N_{01})(N_{00} + N_{01})(N_{00} + N_{10})}},$$

where N_{11}, N_{00}, N_{10}, and $N_{01}$is a number of true positive, true negative, false positive and false negative.

Table S4. Cross tabulation of variants for interaction with NLGN1 and stability change

MCC=0.463		Interaction with NLGN1	
		Decrease	No change
Stability change	Unstable	**T737M**, **D772G**, R813H	M735V, M756I, S763C, S743Y
	No change or more stable		**R856W**, H845Y, T779M, L869M
